# Low Doses of Oxygen Ion Irradiation Cause Acute Damage to Hematopoietic Cells in Mice

**DOI:** 10.1371/journal.pone.0158097

**Published:** 2016-07-01

**Authors:** Jianhui Chang, Yi Luo, Yingying Wang, Rupak Pathak, Vijayalakshmi Sridharan, Tamako Jones, Xiao Wen Mao, Gregory Nelson, Marjan Boerma, Martin Hauer-Jensen, Daohong Zhou, Lijian Shao

**Affiliations:** 1 Division of Radiation Health, Department of Pharmaceutical Sciences, University of Arkansas for Medical Sciences, Little Rock, Arkansas, United States of America; 2 Department of Basic Sciences, Division of Radiation Research, School of Medicine, Loma Linda University, Loma Linda, California, United States of America; ENEA, ITALY

## Abstract

One of the major health risks to astronauts is radiation on long-duration space missions. Space radiation from sun and galactic cosmic rays consists primarily of 85% protons, 14% helium nuclei and 1% high-energy high-charge (HZE) particles, such as oxygen (^16^O), carbon, silicon, and iron ions. HZE particles exhibit dense linear tracks of ionization associated with clustered DNA damage and often high relative biological effectiveness (RBE). Therefore, new knowledge of risks from HZE particle exposures must be obtained. In the present study, we investigated the acute effects of low doses of ^16^O irradiation on the hematopoietic system. Specifically, we exposed C57BL/6J mice to 0.1, 0.25 and 1.0 Gy whole body ^16^O (600 MeV/n) irradiation and examined the effects on peripheral blood (PB) cells, and bone marrow (BM) hematopoietic stem cells (HSCs) and hematopoietic progenitor cells (HPCs) at two weeks after the exposure. The results showed that the numbers of white blood cells, lymphocytes, monocytes, neutrophils and platelets were significantly decreased in PB after exposure to 1.0 Gy, but not to 0.1 or 0.25 Gy. However, both the frequency and number of HPCs and HSCs were reduced in a radiation dose-dependent manner in comparison to un-irradiated controls. Furthermore, HPCs and HSCs from irradiated mice exhibited a significant reduction in clonogenic function determined by the colony-forming and cobblestone area-forming cell assays. These acute adverse effects of ^16^O irradiation on HSCs coincided with an increased production of reactive oxygen species (ROS), enhanced cell cycle entry of quiescent HSCs, and increased DNA damage. However, none of the ^16^O exposures induced apoptosis in HSCs. These data suggest that exposure to low doses of ^16^O irradiation induces acute BM injury in a dose-dependent manner primarily via increasing ROS production, cell cycling, and DNA damage in HSCs. This finding may aid in developing novel strategies in the protection of the hematopoietic system from space radiation.

## Introduction

Radiation is considered to be one of the major risk factors during space activities and has emerged as a critical issue to be resolved for the completion of safe long-duration space missions. The primary components of the space radiation environment are galactic cosmic rays (GCR) and radiation from solar particle events. These space radiation sources consist mainly of protons, helium nuclei and nuclei of elements of atomic number >2 (high-energy high charge particles, HZE), of which ^56^Fe, ^28^Si, ^16^O, and ^12^C are major contributors to dose equivalent in free space. HZE particles are characterized by dense tracks of ionization, a property quantified as high linear energy transfer (LET). While ^56^Fe may be the single largest contributor to GCR dose equivalent in free space[1], space craft material will lead to fragmentation of heavy ions, such that the radiation environment inside a space craft will contain a larger proportion of hydrogen, helium, and ions with smaller masses[2]. ^16^O is representative of the high LET ions inside a space craft. With its relatively high charge and LET it is expected to exhibit a relatively high relative biological effectiveness (RBE). Therefore, we have chosen to investigate the biological effects of ^16^O on cells and tissues in relation to long-duration space missions.

It has been well known that the hematopoietic system is one of the most radiosensitive tissues of the body[3,4]. Total body γ–irradiation (γ–TBI) causes both acute and long-term damage in hematopoietic stem and progenitor cells (HSPCs), which is due primarily to radiation-induced cellular apoptosis and senescence in HSPCs [5,6,7,8]. Total body proton irradiation (proton–TBI) at doses of 0.25–3 Gy significantly decreased the number of white blood cells (WBCs) starting at 4 hours post exposure in a porcine model [9]. In the mouse, proton–TBI results in rapid depletion of peripheral blood WBCs to a minimum at 4 days post irradiation followed by a restoration to near normal levels by two weeks [10]. Two months post proton exposure, the number and function of hematopoietic stem cells (HSCs) in bone marrow (BM) were dramatically impaired, which was mainly mediated by ROS production selectively in HSCs [11]. Moreover, ^56^Fe irradiation at doses of 0.1–0.4 Gy in a mouse model induced significant epigenetic alterations in HSPCs, including methylation of DNA and alterations in the expression of repetitive elements [12]. ^12^C irradiation triggered cellular apoptosis and chromosome aberrations in human HSPCs *in vitro* [13]. These results suggest that various forms of ionizing radiation induce not only acute injury but also long-term damage in hematopoietic cells. However, little information on acute hematopoietic effects of ^16^O exposure is documented.

In the present study, we investigated the acute effects of ^16^O exposure on the hematopoietic system in mice. Specifically, we exposed C57BL/6J mice to 0.1, 0.25 and 1.0 Gy ^16^O (600 MeV/n) total body irradiation (^16^O-TBI) and analyzed the effects of ^16^O irradiation on peripheral blood and BM two weeks after the exposure. Our results showed that exposure to ^16^O decreased the numbers of peripheral WBCs and platelets, and negatively affected the number and function of HPCs and HSCs in mice, coinciding with increased production of reactive oxygen species (ROS), cell cycling and DNA damage.

## Materials and Methods

### Animals and Irradiation

Six-month-old male C57BL/6J mice purchased from the Jackson Laboratory (Bar Harbor, ME) were shipped to Brookhaven National Laboratories (BNL) in Upton, NY. After a one-week acclimation period, the mice were either sham irradiated or received whole-body ^16^O irradiation (600 MeV/n; 0.1, 0.25 and 1.0 Gy, n = 5). For each exposure, animals were individually placed into clear Lucite cubes (3 in x 1½ in x 1½ in) with breathing holes. Sham irradiated mice were placed into the same enclosures for the same amount of time, but were not exposed to radiation. One day after (sham-) irradiation, the mice were shipped to the University of Arkansas for Medical Sciences (UAMS), where they were housed under a constant 12 h light:dark cycle. A standard soy-protein free rodent diet (2020X, Harlan Teklad, Indianapolis, IN) and water were provided *ad libitum*. During the entire experiment, sham-irradiated mice were not housed together with irradiated mice. All procedures in this study were approved by the Institutional Animal Care and Use Committees of the University of Arkansas for Medical Sciences and Brookhaven National Laboratory.

Dosimetry was performed by the NASA Space Radiation Laboratory physics dosimetry group at BNL to ensure the quality of exposure. Briefly, The NASA Space Radiation Laboratory beamline at BNL recorded the charge delivered to a transmission ion chamber placed just in front of the animals. The transmission ion chamber was calibrated against a National Institute of Standards and Technology (NIST)-traceable 1.0 cm^3^ thimble ion chamber (EG&G, Inc.) which was placed at the target position. The doses indicated in the present study were thus the absorbed doses by the animals in the ion chamber at the target surface location on the beamline. The bone marrow of mice is < = 1.0 mm beneath the skin of the animals so the total material between the skin surface and the bone marrow is < 2 mm. With particles of multi-centimeter ranges, there is no significant difference in dose as reported at the surface compared to ~1.5 mm depth for the bone marrow. Therefore, the doses of 0.1, 0.25 and 1.0 Gy ^16^O TBI are the actual doses to the bone marrow in our current study.

### Peripheral Blood and BM Collection

No animal death occurred by two weeks after low doses (0.1, 0.25 and 1.0 Gy) of ^16^O irradiation. Mice were humanely anesthetized and sacrificed with continued exposure to 5% isoflurane for at least 5 min after respiratory arrest at two weeks after radiation exposure, and peripheral blood was collected from the inferior vena cava into EDTA coated tubes. Peripheral blood cell numbers were determined with Vet ABC™ Hematological analyzer (SCIL Animal Care Co.). The femora and tibia were harvested and immediately flushed with Hanks' Balanced Salt Solution (HBSS) containing 2% fetal bovine serum (FBS) using a 21-gauge needle and syringe to collect the BM. BM samples were placed on ice and analyzed within the same day as described below.

### Isolation of BM Mononuclear Cells (BM-MNCs), Analysis of the Frequencies and Numbers of Different Hematopoietic Cell Populations by Flow Cytometry

BM cells were pre-incubated with biotin-conjugated anti-CD3e, anti-CD45R/B220, anti-Gr-1, anti-CD11b, and anti-Ter-119 antibodies and with anti-CD16/32 (Fcγ II/III Receptor or FcγR) antibody to block the Fcγ receptors. Cells were then labeled with streptavidin–FITC, anti-Sca-1–PE-Cy7, anti-c-Kit–APC-Cy7, anti-CD150-APC and anti-CD48-Pacific blue for HPCs (Lin^-^Sca1^-^c-kit^+^ cells), LSK cells (Lin^-^Sca1^+^c-kit^+^ cells), and HSCs (Lin^-^Sca1^+^c-kit^+^CD150^+^CD48^-^ cells). BM-MNCs were isolated by Histopaque 1083 separation solution (Sigma, St. Louis, MO). For the isolation of Lineage negative cells (Lin^-^ cells), BM-MNCs were incubated with purified rat antibodies specific for murine CD3e, Mac-1, CD45R/B220, Ter-119, and Gr-1. The labeled mature lymphoid and myeloid cells were depleted by incubating with goat anti-rat IgG paramagnetic beads (Life Technologies, Grand Island, NY) at a bead:cell ratio of approximately 4:1. Cells binding the paramagnetic beads were removed with a magnetic field. Lin^-^ cells were washed twice with 2% FBS/HBSS and re-suspended in complete medium (RPMI1640 medium supplemented with 10% FBS, 2 mM L-glutamine, 10 μM HEPES buffer, and 100 U/mL penicillin and streptomycin) at 1x10^7^ cells/mL. Subsequently, cells were blocked by Fcγ receptors anti-CD16/32 antibody and then stained with anti-Sca1-PE-Cy7, c-Kit-APC-Cy7. All flow antibodies were purchased from eBioscience (San Jose, CA). The frequencies of HPCs and HSCs were analyzed with an Aria II cell sorter. Dead cells were excluded by gating out the cells stained positive with propidium iodide (PI). For each sample, approximately 8 x 10^5^ to 1 x 10^6^ BM cells were acquired and the data were analyzed using BD FACSDiva 6.0 (BD Biosciences) and FlowJo (FlowJo, Ashland, OR) software.

### Colony-forming Cell (CFC) and Cobblestone Area-forming Cell (CAFC) Assays

The CFC assay was performed by culturing BM-MNCs in MethoCult GF M3434 methylcellulose medium (Stem Cell Technologies, Vancouver, BC). Colonies of CFU–granulocyte macrophage (GM) and burst-forming unit–erythroid (BFU-E) were scored on day 7, and colonies of CFU-granulocyte, -erythrocyte, -monocyte, and -megakaryocyte (GEMM) on day 12 of the incubation, according to the manufacturer’s protocol. The CAFC assay was performed as described elsewhere [8,14].

### Analysis of the Levels of Intracellular ROS

After staining with the appropriate cell surface marker antibodies, Lin^-^ cells (1 x 10^7^/mL) were suspended in PBS supplemented with 5 mM glucose, 1 mM CaCl_2_, 0.5 mM MgSO_4_, and 5 mg/ml BSA and then incubated with 10 μM 2',7'-dichlorofluorescein diacetate (DCFDA) (Life Technologies, Grand Island, NY) for 30 minutes at 37°C. The levels of ROS in HPCs and HSCs were analyzed by measuring the mean fluorescence intensity (MFI) of 2',7'-dichlorofluorescein (DCF) with an Aria II cell sorter. For each sample, a minimum of 200,000 lineage negative cells was acquired and the data were analyzed as previously described [7].

### DNA Damage Analysis

Lin^-^ cells were first stained with antibodies against various cell-surface markers and fixed and permeabilized using the Fixation/Permeabilization Solution from BD Biosciences (San Diego, CA) followed by 0.2% Triton-X-100 incubation for 10 min. Cells were then stained with Alexa Fluor 488 conjugated anti-phospho-Histone 2AX (Ser139) antibody for 1.5 hours at 4°C and analyzed by flow cytometry. The levels of DNA double strand break damage were expressed by the mean fluorescence intensity of phospho-Histone 2AX (γH2AX) with an Aria II cell sorter.

Approximately 4000 sorted HPCs and HSCs were cytospun onto slides and fixed in 4% paraformaldehyde solution for 10 min at room temperature. Cells were permeabilized with 0.2% Triton X-100 on ice and blocked with 5% fetal bovine serum (FBS) before incubation with anti-γH2AX (1:1000, Millipore, Billerica, MA) overnight at 4°C. Cells were then treated with Alexa Fluor-488-conjugated anti-mouse IgG antibody (1:1000, Life Technologies, Grand Island, NY) in 5% FBS solution for 1 h at room temperature. Nuclei were counterstained with Hoechst-33342. Slides were finally mounted in Vectashield (Vector Laboratories, Burlingame, CA). Images were acquired using fluorescence microscopy. Approximately 100 nuclei images were acquired using a fluorescence microscope and the number of γH2AX foci for each cell was counted, averaged, and expressed as γH2AX foci/cell.

### Cell Cycle Analysis

Lin^-^ cells were first stained with antibodies against various cell-surface markers and fixed and permeabilized using the Fixation/Permeabilization Solution (BD Biosciences). Subsequently, they were stained with anti-Ki67-FITC antibody (BD Biosciences) and 7-aminoactinomycin (7-AAD, Sigma, St. Louis, MO) and then analyzed by flow cytometry.

### Statistical Analysis

All data are presented as mean ± standard deviation of the mean of at least five independent biological samples per radiation dose. The differences between sham-irradiated and irradiated groups were examined by one-way ANOVA. The differences in the distribution of the cell cycle phases were determined by Chi-Square test. Differences were considered significant at *p* < .05. Statistical analysis was performed using GraphPad Prism (GraphPad Software Inc. LaJolla, CA).

## Results

### ^16^O Irradiation Decreased Blood Cell Counts

**Fig 1:**
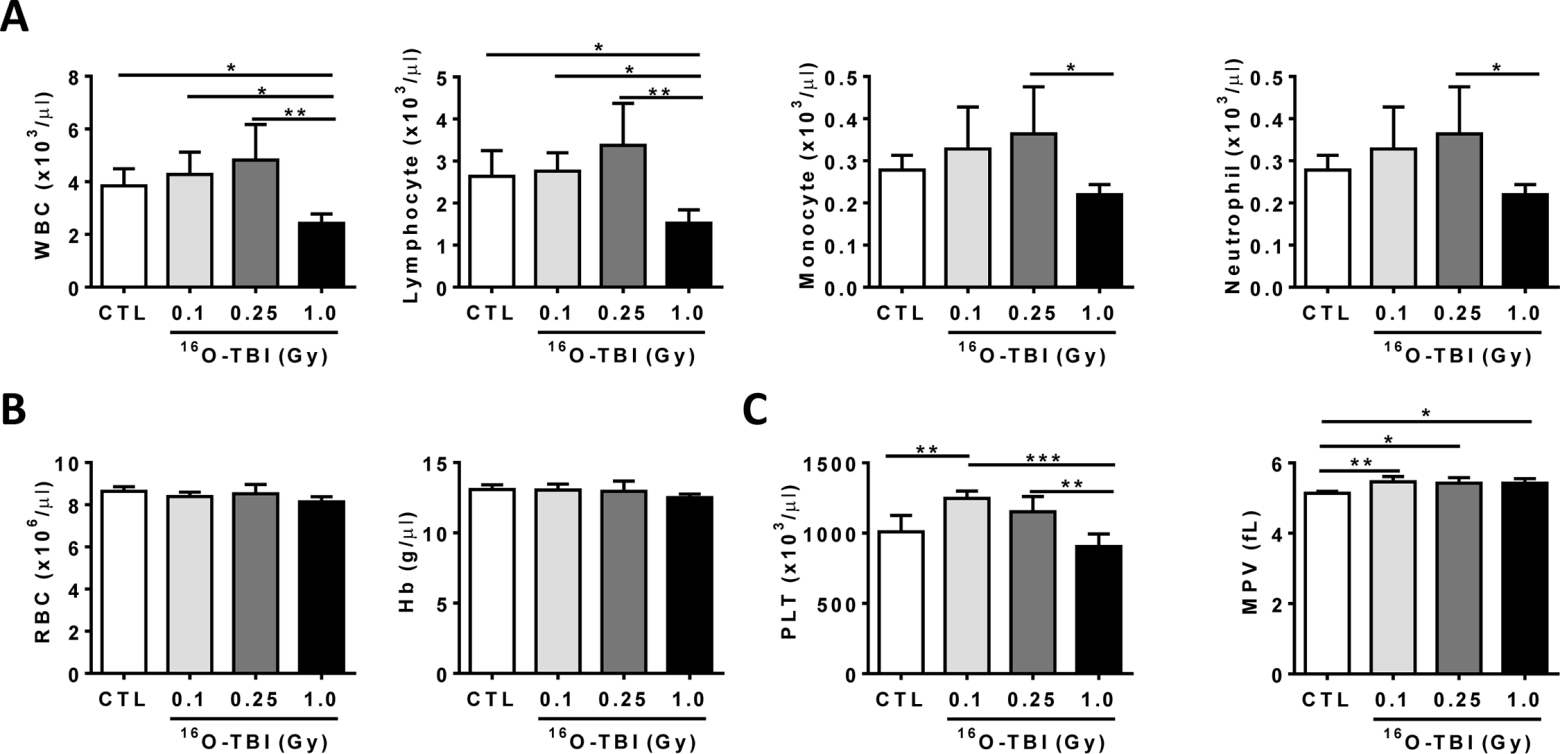
Blood cell counts were decreased under 1.0 Gy ^16^O exposure. C57BL/6J mice were exposed to 0.1, 0.25 and 1.0 Gy doses of ^16^O irradiation or were sham irradiated as a control (CTL). The cell counts in peripheral blood were determined two weeks after radiation exposure. (A) The numbers of WBC, lymphocytes, monocytes, and neutrophils were significantly reduced after a dose of 1.0 Gy only. (B) The levels of RBC and Hb after 0.1, 0.25 and 1.0 Gy doses exposure were comparable to those in CTL mice. (C) The platelet counts (PLT) and mean platelet volume (MPV) were affected by all doses of ^16^O. The statistical significance for the difference between the control group and each of the irradiated groups is indicated by asterisks: *p<0.05, **p<0.01, ***p<0.001 as determined by one-way ANOVA.

Previous reports showed that peripheral blood cell counts were decreased up to at least three weeks after exposure of mice to 230 MeV protons [15]. We thus measured blood cell counts two weeks after ^16^O exposure as shown in Fig 1. The data revealed that peripheral WBC and lymphocyte counts were significantly reduced at two weeks after 1.0 Gy ^16^O exposure compared to non-irradiated mice (Fig 1A, *p*<0.05-*p*<0.01). However, there appeared to be an increased trend of WBC, lymphocyte, monocyte and neutrophil counts without statistical significance after 0.1 and 0.25 Gy of ^16^O-TBI in comparison to control animals (Fig 1A, *p*>0.05). The counts of WBCs, lymphocytes, monocytes, neutrophils and platelets showed significant decrease after 1.0 Gy ^16^O-TBI compared to 0.25 Gy radiation (Fig 1A and 1C, *p*<0.05-*p*<0.01), while no differences in red blood cell (RBC) counts and hemoglobin (Hb) were observed after any of the doses used (Fig 1B, p>0.05). There was an increased platelet count after 0.1 Gy ^16^O-TBI compared to non-irradiated and 1.0 Gy irradiated mice (Fig 1C, *p*<0.01-*p*<0.001). Mean platelet volume (MPV) after all three different doses of ^16^O-TBI was slightly but significantly increased compared to control mice (Fig 1C, *p*<0.05-*p*<0.01).

### ^16^O Irradiation Induced HPC and HSC Injuries

**Fig 2:**
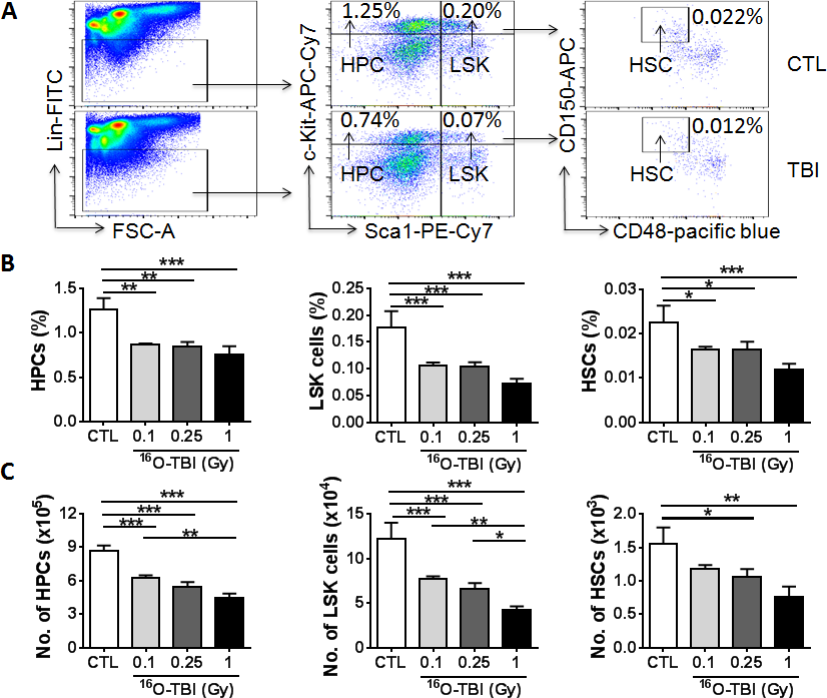
^16^O TBI causes reductions in numbers of HPCs and HSCs at two weeks after irradiation in a radiation dose dependent manner. (A) Representative gating strategy of flow cytometric analysis for HPCs (Lin^-^Sca1^-^c-kit^-^ cells), LSK cells (Lin-Sca1^+^c-kit^+^cells) and HSCs (Lin^-^Sca1^+^c-kit^+^CD150^+^CD48^-^ cells) in BMCs is shown from 1.0 Gy ^16^O TBI and sham-irradiation (CTL). (B and C) Frequencies (panel B) and total numbers (panel C) of HPCs, LSK cells and HSCs from each mouse are presented as mean ± SD (n = 5). The statistical significance for the difference between the control group and each of the irradiated groups is indicated by asterisks. *p<0.05, **p<0.01, ***p<0.001 as determined by one-way ANOVA.

Mature peripheral blood cells are derived from HSCs through multipotent progenitor cell differentiation. It is unclear whether the abnormalities in peripheral blood are induced by ^16^O irradiation due to a defect in HSCs. To test this possibility, two weeks after ^16^O TBI we analyzed the frequencies and numbers of different hematopoietic cell populations in BM cells by flow cytometry as shown in Fig 2A. The results demonstrated that all doses of ^16^O significantly reduced both the frequencies of HPCs (Lin^-^Sca1^-^c-kit^+^ cells), LSK (Lin^-^Sca1^+^c-kit^+^ cells) and HSCs (Lin^-^Sca1^+^c-kit^+^CD150^+^CD48^-^ cells) (Fig 2B, *p*<0.05-*p*<0.001) and their total numbers (Fig 2C, *p*<0.05-*p*<0.001) in a dose-dependent manner.

**Fig 3:**
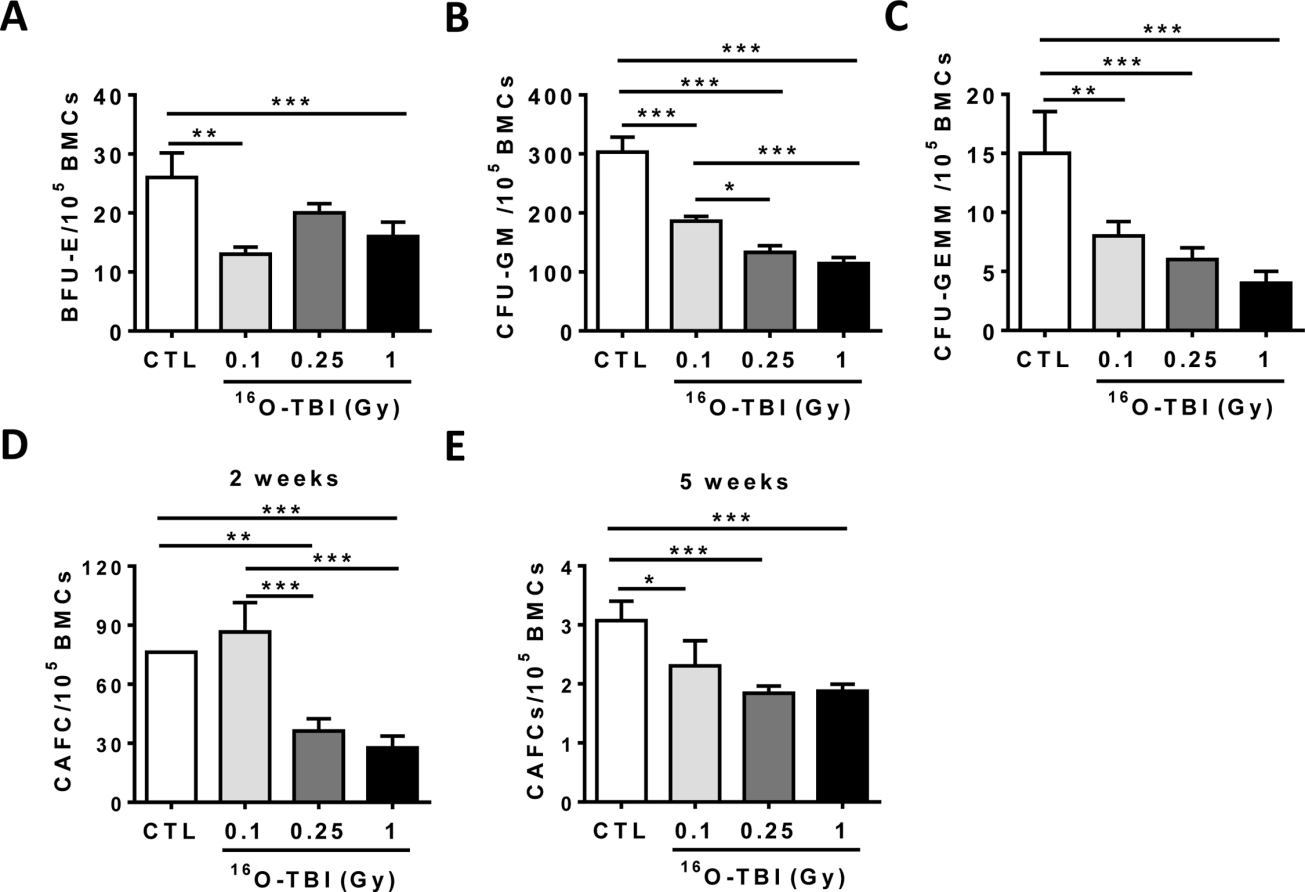
^16^O TBI causes sustained reduction of HSC clonogenic function. (A–C) At 2 weeks after TBI, BM-MNCs were isolated from irradiated and sham-irradiated (CTL) mice. A CFC assay was performed, and the results are presented as mean CFUs per 1x10^5^ BM-MNCs (n = 5). (D and E) Total BM cells (BMCs) were analyzed by CAFC assays, and the numbers of two and five week CAFCs were expressed as mean ± SD (n = 3 mice per group) per 1x10^5^ BMCs. The statistical significance for the difference between the control group and each of the irradiated groups is indicated by asterisks. *p<0.05, **p<0.01, ***p<0.001 as determined by one-way ANOVA.

We further examined whether ^16^O irradiation may not only alter the frequency and number, but also the function of HPCs and HSCs. Firstly, we used a colony forming unit assay and showed that the frequencies of BFU-Es, CFU-GMs, and CFU-GEMMs were significantly reduced in all irradiated mice compared to those in sham-irradiated controls (Fig 3A–3C, *p*<0.05-*p*<0.001), indicating that the abilities of hematopoietic progenitor cells (HPCs) to differentiate into granulocytes, erythrocytes, monocytes and/or megakaryocytes were dramatically decreased upon ^16^O exposure. Hematopoietic stem cells (HSCs) were then analyzed with a CAFC assay, a widely used *in vitro* surrogate assay of HSC function. The frequencies of functional HSCs at 2- and 5-week CAFCs were significantly lower in irradiated mice, particularly in the 0.25 and 1.0 Gy groups (Fig 3D and 3E, *p*<0.05-*p*<0.001). These findings clearly demonstrate that exposure to ^16^O irradiation causes acute damage to HPCs and HSCs.

### ^16^O Irradiation Induced Oxidative Stress in HPCs and HSCs

**Fig 4:**
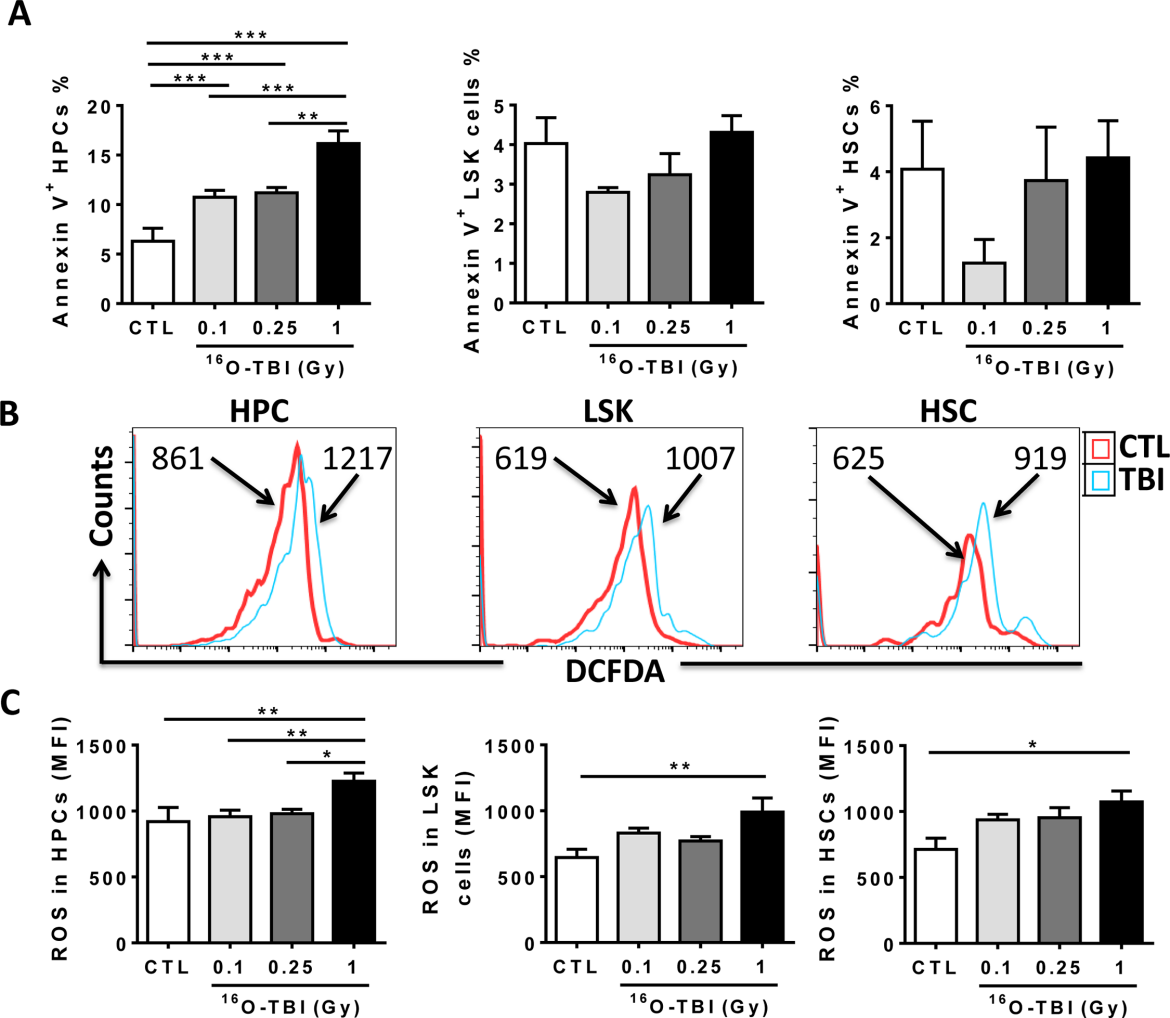
^16^O TBI causes an increase in ROS production in HPCs and HSCs two weeks after 1.0 Gy exposure and an increase in apoptosis in HPCs. (A) Isolated Lin^-^ cells were stained with Annexin V to determine cellular apoptosis, and percentages of Annexin V positive cells were presented as mean ± SD (n = 5). (B) Representative analysis of ROS production as measured by flow cytometry using DCFDA in BM HPCs and HSCs from control and 1.0 Gy^16^O TBI mice. The histograms indicate DCF MFI from a representative experiment. (C) The DCF MFI in BM HPCs and HSCs are presented as mean ± SD (n = 5). The statistical significance for the difference between the control group and each of the irradiated groups is indicated by asterisks. *p<0.05, **p<0.01, ***p<0.001 as determined by one-way ANOVA.

According to data from mouse models of γ-irradiation, the acute effects of γ-rays are primarily attributed to cell apoptosis in progenitors and stem cells [5,6]. We thus measured apoptosis in BM cells from ^16^O exposed mice, using Annexin V staining (Fig 4A). Compared to non-irradiated controls, 0.1 and 0.25 Gy ^16^O irradiation significantly increased the ratio of apoptotic cells in HPCs, while 1.0 Gy ^16^O irradiation had a much greater effect on apoptosis in HPCs but not in LSK and HSCs. Notably, the amount of apoptotic cells in both LSK and HSC populations was decreased after 0.1 Gy ^16^O-TBI compared to control cells even though it didn’t reach statistical significance using one-way ANOVA analysis.

Apart from apoptosis, oxidative stress might play an important role in HSC deficiency under radiation conditions. Our previous studies have revealed that γ-irradiation induced long-term HSC damage primarily via induction of increased production of ROS and chronic oxidative stress [7]. We therefore examined intracellular production of ROS in different populations of hematopoietic cells in BM from ^16^O-irradiated mice. As shown in Fig 4B and 4C, ROS production in HPCs and HSCs from 1.0 Gy irradiated mice was higher than that in cells from 0.1 Gy, 0.25 Gy, and sham-irradiated mice (*p*<0.05-*p*<0.01). Exposure of mice to 0.1 and 0.25 Gy TBI showed a trend to increased levels of ROS in HSCs without reaching statistical significance.

### ^16^O Irradiation Induced Cell Cycling and DNA Damage in HPCs and HSCs

**Fig 5:**
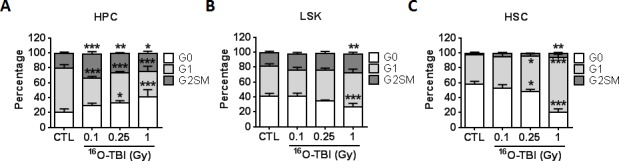
^16^O TBI drives HSCs from quiescence into the cell cycle. Lin^−^cells were isolated from control (CTL) and irradiated (TBI) mice 2 weeks after 0.1, 0.25 and 1.0 Gy TBI. Cell cycle was measured by flow cytometry using Ki-67 and 7-AAD double staining in BM HPCs and HSCs from control and irradiated mice. (A-C) The percentages of G0, G1 and G2SM phases in BM HPCs, LSK cells and HSCs after TBI are presented as mean ± SD (n = 5). The distribution of cell cycle phases in HPCs, LSK cells and HSCs was analyzed by Chi-Square test as indicated by X^2^ (HPC, X^2^ = 19.084, p<0.05; LSK cells, X^2^ = 6.486, p<0.05; HSCs, X^2^ = 33.853, p<0.05). The statistical significance for the difference in each cell cycle phase between the control groups and irradiated groups is indicated by asterisks. *p<0.05, **p<0.01, ***p<0.001 by one-way ANOVA analysis.

Increased ROS production can stimulate stem cell cycling and cause oxidative DNA damage, resulting in stem cell exhaustion and induction of stem cell senescence. To investigate the cell cycle status of HPCs and HSCs, Ki-67 and 7-AAD double staining was utilized (Fig 5). Overall, the distribution of cell cycle phases in irradiated HPCs, LSK cells and HSCs was significantly different from that in non-irradiated counterpart populations (HPCs, X^2^ = 19.084, p<0.05; LSK cells, X^2^ = 6.486, p<0.05; HSCs, X^2^ = 33.853, p<0.05). Intriguingly, in irradiated mice, higher numbers of HPCs were in G_0_ and G_2_SM phases and lower numbers were in the G_1_ phase compared to the cell cycle distribution of HPCs in sham-irradiated mice (Fig 5A). As expected, the increased production of ROS in irradiated HSCs was associated with a significant reduction in LSK and HSC quiescence (fewer G_0_ phase cells p<0.05 and more G_1_ and G_2_SM phase cells, *p*<0.05-*p*<0.001) in a dose-dependent manner, indicating that ^16^O irradiation stimulated LSK and HSC cycling and proliferation (Fig 5B and 5C). Increased cycling of HSCs after ^16^O irradiation may compensate for the decrease of HSCs but at the expense of HSC self-renewal, which can result in the damage to the hematopoietic system.

**Fig 6:**
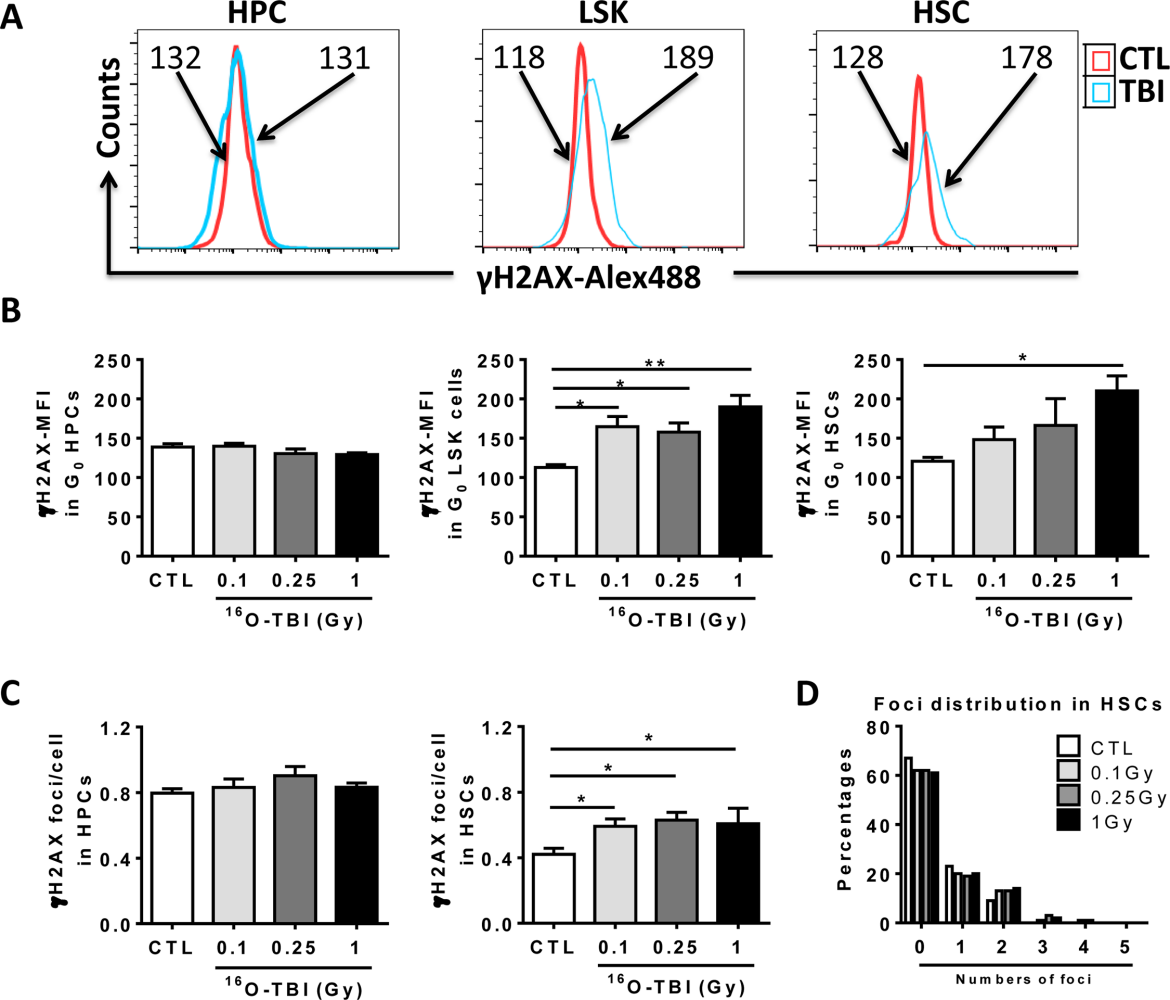
^16^O TBI causes persistent increases in DNA damage in HSCs but not in HPCs two weeks after the exposure. (A) Representative analysis of DNA damage measured in Lin^-^ cells by flow cytometry using γH2AX staining in BM HPCs and HSCs from control and 1.0 Gy^16^O TBI mice. The histograms indicate γH2AX MFI from a representative experiment. (B) The γH2AX MFI in BM HPCs and HSCs after TBI are presented as mean ± SD. (C) Sorted HPCs and HSCs from irradiated and non-irradiated mice were stained with γH2AX antibody. The numbers of foci in each cell were counted and expressed as mean ± SD. (D) The distribution of foci was expressed as the percentages of different numbers of foci in control and irradiated HSCs. The statistical significance for the difference between the control groups and each of irradiated groups is indicated by asterisks. *p<0.05, **p<0.01 by one-way ANOVA analysis.

If ^16^O TBI can induce oxidative stress in HPCs and HSCs, this may also cause DNA damage, known to impair HPC and HSC function. To test this hypothesis, we used flow cytometry to analyze mean fluorescence intensity (MFI) of γH2AX immunostaining in HPCs and HSCs as an indication of DNA double strand breaks (Fig 6A). HPCs from irradiated mice showed no significant change in MFI of γH2AX staining (Fig 6B). However, a significant increase in γH2AX MFI was observed in LSK after all doses of ^16^O, and in HSCs after the 1.0 Gy dose (Fig 6B, *p*<0.05-*p*<0.01). To confirm ^16^O irradiation-induced DNA damage in HPCs and HSCs, we isolated HPCs and HSCs from irradiated and non-irradiated mice by cell sorting and conducted γH2AX immunostaining. The number of γH2AX foci in each cell was counted under a fluorescent microscope. The data indicated that irradiated HSCs, but not irradiated HPCs, had significantly higher numbers of γH2AX foci per cell compared to non-irradiated HSCs (Fig 6C, *p*<0.05), which is consistent with the data from MFI of γH2AX by flow cytometry. Moreover, we further analyzed the foci distribution in HSCs. The results showed that more than 60% of HSCs from irradiated and non-irradiated mice did not have γH2AX foci. About 15–17% of HSCs contained more than two γH2AX foci in each HSC from irradiated mice while only 9% of normal HSCs had two foci per cell (Fig 6D). Therefore, these data demonstrate that ^16^O irradiation induces DNA damage in irradiated LSK and HSCs, which may contribute to ^16^O irradiation-induced acute hematopoietic damage.

## Discussion

The data presented here are the first to report acute damage in HPCs and HSCs in C57BL/6J mice after whole body exposure to 600 MeV/n ^16^O at a dosage range relevant to what is found in space. We utilized the actual absorbed doses (0.1, 0.25 and 1.0 Gy) by bone marrow cells to investigate the side effects of ^16^O exposure to hematopoietic cells, which is also beneficial to our data translation to human hematopoietic studies in the future. Our data indicated that the negative changes in function of HPCs and HSCs coincided with radiation-induced oxidase stress, as indicated by increased levels of ROS in these cells after 1.0 Gy of ^16^O exposure but not 0.1 and 0.25 Gy. Because these effects were seen at two weeks after 1.0 Gy of ^16^O exposure, our results suggest that a persistent production of ROS had occurred in HSCs. Of note, the increased ROS production may have been the cause of the observed increase in DNA damage and stimulated the entry of quiescent HSCs into the cell cycle. Some HSC features include slow cycling and quiescence, dividing only once per 145 days, which contributes to the high self-renewal potential of HSCs. The cell cycle re-entry induced by ^16^O irradiation may have been an attempt to compensate for the decrease of HSCs at the expense of their self-renewal in irradiated mice.

Since hematopoietic cells are known to be radiosensitive, it is not surprising that a significant decrease was observed in peripheral WBC and lymphocyte counts in mice exposed to 1.0 Gy of ^16^O. However, exposure of 0.1 and 0.25 Gy of ^16^O TBI to mice induced an increase in peripheral WBC, lymphocyte, monocyte and neutrophil counts compared to non-irradiated mice. The responses to the two lower doses also differed from the responses to 1.0 Gy of ^16^O TBI through increased platelet numbers, no increase in ROS in HPCs and, for 0.1 Gy, a decrease in HSC apoptosis. This may indicate different mechanisms induced by 0.1 and 0.25 Gy exposure compared to 1.0 Gy exposure, consistent with the observations of adaptive responses, hyper-radiosensitivity and bystander effects after γ-irradiation at low doses [16,17,18]. Previous studies have shown that the numbers of peripheral WBCs and platelets returned to normal levels at two weeks after exposure of BALB/c mice to 1.0 Gy of γ-ray irradiation [19]. The differences between ^16^O and γ-ray irradiation may be due to the high LET properties of ^16^O. There was an increased apoptosis in HPCs, but not HSCs 2 weeks after 1.0 Gy of ^16^O TBI, which might result in the decrease of HPC colony forming abilities along with fewer numbers of BFU-E, CFU-GM and CFU-GEMM compared to those in control HPCs. To further answer whether various hematopoietic cell populations can recover from ^16^O TBI-induced apoptosis, we performed additional apoptotic assay in HPCs, LSK cells and HSCs at 3 months after the same doses of ^16^O TBI. Our data showed that the apoptotic levels in HPCs, LSK cells and HSCs were all back to normal at 3 months after ^16^O-TBI (data not shown), indicating that HPCs exhibited a slower recovery from ^16^O-TBI-induced apoptosis than HSCs. Previous studies have shown that 1.0 Gy of γ-ray irradiation dramatically impaired the engraftment ability of HSCs in mice [19, 20]. Moreover, the number of mouse HSCs was significantly decreased at day 10 after a single dose of 5-Fluorouracil treatment [21]. These data indicate that mouse HSCs are highly sensitive to radio- and chemo-therapy administration. Using *in vitro* colony forming assays, our current study is the first to demonstrate that ^16^O exposure not only reduced the numbers of HSCs, but also their function, indicating that HSCs are sensitive to ^16^O irradiation at total doses of 0.1–1.0 Gy. We previously reported that exposure of mice to 6.5 Gy total body γ-irradiation decreased the numbers of HSCs by 50% at 2 weeks post exposure [8]. Our data presented here showed that the numbers of HSCs were reduced by 50% at 2 weeks after 0.25 and 1.0 Gy total body ^16^O irradiation. In addition, ^16^O irradiation has more significant effects on cell survival, micronuclei formation, chromosomal aberrations, and apoptosis compared to photon irradiation in cell cultures [22,23]. Overall, these data confirm the characteristic of a high RBE for ^16^O.

In this study, an aberrant increase in ROS production was detected in the HPC and HSC compartments after a dose of 1.0 Gy of ^16^O. However, we detected the increased levels of apoptosis in irradiated HPCs but not LSK cells and HSCs. These differences between HPCs and HSCs could not be attributable to oxidative stress. The underlying causes of these differences have yet to be studied. The deleterious impact of ROS accumulation in HSCs has been extensively investigated using γ–irradiation and genetic knockout mice. For example, we have previously shown that sublethal doses of γ–rays induce increased levels of ROS in HSCs in a mouse model [7,14]. Other reports have demonstrated that the increased ROS production in Foxo3, ATM, TSC1 and Bmi-1 mutant mice contributed to the impairment in number and function of HSCs, and that antioxidants such as N-acetyl cysteine (NAC) could attenuate the HSC defect in these mutant mice [24,25,26,27]. However, little is known about the role of oxidative stress, the source of ROS, and the signaling pathways governing the regulation of ROS in hematopoietic stem cells after ^16^O irradiation.

Mitochondrial oxidative phosphorylation and NADPH oxidases (NOXs) are the two main drivers to generate ROS in mammalian cells. Production of ROS in HSCs is unlikely derived from mitochondrial oxidative phosphorylation, because of the localization of HSCs in hypoxic bone marrow niches and the high levels of hypoxia inducible factor 1α in HSCs. We recently revealed that NADPH oxidase 4 (NOX4) expression was significantly up-regulated in HSCs from proton and γ-ray irradiated mice [11,28]. Moreover, the NOX4 inhibitor diphenyliodonium ameliorated γ-irradiation-induced ROS production in HSCs and partially restored HSC function. Our current data showed that 1.0 Gy of ^16^O irradiation induced ROS production in not only HPCs but also HSCs, suggesting that oxidative stress may play a role in ^16^O irradiation-induced hematopoietic cell damage. Therefore, future studies should test whether utilization of anti-oxidants, such as NOX4 inhibitors and NAC, may reduce ^16^O irradiation-induced ROS production to improve the function of HPCs and HSCs after ^16^O irradiation.

Maintaining proper HSC function mainly depends on keeping these cells quiescent and maintaining their abilities for self-renewal [29,30]. Under stress conditions, quiescent HSCs could be activated to undergo proliferation, mobilization and differentiation to compensate for the hematopoietic stress. Our previous studies have shown that proton and γ-irradiation activated HSCs [7,11], reducing the numbers of HSCs in the G_0_ phase and increasing numbers of HSCs in the G_1_ phase compared to non-irradiated controls. This imbalance may lead to the abnormalities in HSC self-renewal and colony forming ability. It has also been shown that mutations of FOXO3a and Lkb1 in mice accelerates HSC cycling, which eventually causes the loss of stem cell self-renewal ability and the exhaustion of HSCs [24,31]. Consistently, ^16^O exposure at doses of 0.25 and 1.0 Gy stimulated quiescent HSCs enter into the cell cycle, resulting in fewer numbers of HSCs in the G_0_ phase and higher numbers in the G_1_/G_2_SM phases, particularly accumulating in the G1 phase. This would insure that there were enough cycling HSCs to compensate for the loss of HSCs after ^16^O irradiation, while it resulted in the impairment of HSC self-renewal ability, evidenced by fewer numbers of CAFC formation. In addition, we analyzed the levels of DNA damage in different cell cycle phases in HPCs and HSCs, demonstrating that ^16^O irradiation induced persistent increases in DNA damage selectively in G0 phase of HSCs but not in G1/SG2M phases (data not shown). However, this phenomenon wasn’t found in the HPC population after ^16^O exposure, indicating that there are different underpinning mechanisms in the impairment of HPCs and HSCs under ^16^O irradiation. DNA double strand breaks are usually repaired quickly and γH2AX foci begin to disappear with 1–4 hours with significant depletion 24 hours post radiation exposure. We further analyzed the DNA damage in sorted HSCs using γH2AX immunostaining and demonstrated that more than 15% of irradiated HSCs contained more than two γH2AX foci per cell 2 weeks after ^16^O TBI. These data are positively correlated with the increased oxidative stress in irradiated HSCs. We therefore suspect that continual ROS production may be responsible for persistent DNA damage in HSCs post ^16^O exposure. To test this possibility, we will treat mice with antioxidants after irradiation and check the levels of DNA damage in non-irradiated and irradiated HSCs in our future studies.

In summary, our study provides important biological data indicative of oxidative stress and altered function in hematopoietic progenitor and stem cells at two weeks after exposure to ^16^O in a mouse model. These results may have important implications for health outcomes during long-duration space flights. Knowledge gained from this study could also aid in planning countermeasure strategies to protect against hematopoietic effects of radiation exposure during space travel.
